# Molecular basis for impaired collateral artery growth in the spontaneously hypertensive rat: insight from microarray analysis

**DOI:** 10.1002/phy2.5

**Published:** 2013-06-26

**Authors:** Joseph L Unthank, Jeanette N McClintick, Carlos A Labarrere, Lang Li, Matthew R DiStasi, Steven J Miller

**Affiliations:** 1 Department of Surgery, Indiana University School of MedicineIndianapolis, Indiana, 46202; 2 Department of Cellular and Integrative Physiology, Indiana University School of MedicineIndianapolis, Indiana, 46202; 3 Indiana Center for Vascular Biology and Medicine, Indiana University School of MedicineIndianapolis, Indiana, 46202; 4 Department of Biochemistry and Molecular Biology, Indiana University School of MedicineIndianapolis, Indiana, 46202; 5 Center for Medical Genomics, Indiana University School of MedicineIndianapolis, Indiana, 46202; 6 Division of Experimental Pathology, Indiana University Health, Methodist HospitalIndianapolis, Indiana, 46202; 7 Center for Computational Biology and Bioinformatics, Indiana University School of MedicineIndianapolis, Indiana, 46202; 8 Department of Microbiology and Immunology, Indiana University School of MedicineIndianapolis, Indiana, 46202

**Keywords:** Arteriogenesis, collateral gene expression, microarray analysis, peripheral vascular disease

## Abstract

Analysis of global gene expression in mesenteric control and collateral arteries was used to investigate potential molecules, pathways, and mechanisms responsible for impaired collateral growth in the Spontaneously Hypertensive Rat (SHR). A fundamental difference was observed in overall gene expression pattern in SHR versus Wistar Kyoto (WKY) collaterals; only 6% of genes altered in collaterals were similar between rat strains. Ingenuity® Pathway Analysis (IPA) identified major differences between WKY and SHR in networks and biological functions related to cell growth and proliferation and gene expression. In SHR control arteries, several mechano-sensitive and redox-dependent transcription regulators were downregulated including JUN (−5.2×, *P* = 0.02), EGR1 (−4.1×, *P* = 0.01), and NFĸB1 (−1.95×, *P* = 0.04). Predicted binding sites for NFĸB and AP-1 were present in genes altered in WKY but not SHR collaterals. Immunostaining showed increased NFĸB nuclear translocation in collateral arteries of WKY and apocynin-treated SHR, but not in untreated SHR. siRNA for the p65 subunit suppressed collateral growth in WKY, confirming a functional role of NFkB. Canonical pathways identified by IPA in WKY but not SHR included nitric oxide and renin–angiotensin system signaling. The angiotensin type 1 receptor (AGTR1) exhibited upregulation in WKY collaterals, but downregulation in SHR; pharmacological blockade of AGTR1 with losartan prevented collateral luminal expansion in WKY. Together, these results suggest that collateral growth impairment results from an abnormality in a fundamental regulatory mechanism that occurs at a level between signal transduction and gene transcription and implicate redox-dependent modulation of mechano-sensitive transcription factors such as NFĸB as a potential mechanism.

## Introduction

An urgent medical need has been identified for novel therapies across the spectrum of peripheral arterial disease (PAD) (Gornik 2009), the prevalence of which is rising dramatically with increased occurrence of risk factors including aging, obesity, and hyperglycemia. These risk factors for PAD not only promote arterial disease, but also suppress the innate capacity for compensation to major arterial occlusion in humans and animal models (as reviewed by Ziegler et al.^2010^). Their presence may also suppress the efficacy of therapeutic interventions, including molecular and cell based treatments (Kinnaird et al. 2008) and thus explain the failure of many preclinical studies performed in young healthy animals to predict clinical outcomes for PAD therapies. While potential mechanisms that mediate impaired compensation have been reviewed (Kinnaird et al. 2008), many of the studies supporting these potential mechanisms were focused on angiogenesis or tissue/cellular responses to severe ischemia or hypoxia and not the primary collateral arteries which experience increased blood flow and shear stress and comprise the primary site of vascular resistance and compensation subsequent to arterial occlusion in both humans and animal models of arterial insufficiency (as reviewed in Ziegler et al. 2010). Consequently, the specific mechanisms mediating the impaired growth of collateral arteries in the peripheral circulation remain largely unknown.

The current study was undertaken to identify potential mechanisms responsible for impaired collateral growth in the Spontaneously Hypertensive Rat (SHR), an animal model widely utilized to assess vascular adaptations to peripheral arterial occlusion (Nelissen-Vrancken et al. 1992, 1993; Scheidegger et al. 1997; Emanueli et al. 2001, 2002; Tamarat et al. 2002; Tuttle et al. 2002b; Srivastava et al. 2003; Iaccarino et al. 2005; Miller et al. 2007a; You et al. 2008; Matsumura et al. 2009). Microarray analysis of control and collateral mesenteric arteries was performed and the results demonstrate a fundamental difference in the overall collateral gene expression pattern in SHR versus normotensive control rats (Wistar Kyoto; WKY). Data mining revealed striking differences in the expression of molecules involved in the regulation of cell growth and proliferation and gene expression. Redox-dependent modulation of mechano-sensitive transcription factors is proposed as a potential mechanism that may explain, at least in part, the fundamental differences in collateral gene expression between WKY and SHR and the resultant impairment in SHR collateral growth.

## Methods

### General experimental approach

The mesenteric model of flow-mediated collateral growth (Unthank et al. 1996a,b) was used to assess mechanisms related to collateral growth and its impairment. The mesentery is part of the peripheral circulation and both acute and chronic intestinal occlusion occur clinically as a result of atherosclerotic lesions (Hirsch et al. 2006). This model allows similar ileal arteries representing normal flow control arteries and high flow collateral arteries to be evaluated in the same animal (within subject design). Additional advantages of the mesenteric model include a well-defined collateral path and known hemodynamic changes (Unthank et al. 1996a,b). The vessels are of a size that provide significant compensation as primary collaterals, can be easily dissected, and provide adequate RNA for analysis without pooling.

### Animals, procedures, and treatments

Male WKY and SHR rats were obtained from Harlan (Indianapolis, IN) and studied at ∼10 weeks of age. All procedures were approved by the Indiana University School of Medicine Institutional Animal Care and Use Committee. The mesenteric model to induce collateral growth was created by ligation of sequential ileal arteries with special care to prevent angiogenesis from stretch or partial desiccation as previously described (Unthank et al. 1996a,b; Miller et al. 2007a).

For microarray analysis and real-time quantitative RT-PCR studies, control and collateral arteries were harvested 24 h postligation. This time was selected because minimal cellular remodeling/recruitment and luminal expansion have occurred and previous array and expression studies have shown significant mRNA alterations at 24 h after arterial occlusion (Lee et al. 2004; Prior et al. 2004). Prior to tissue harvesting, the caudal aorta was cannulated above the iliac bifurcation, and the renal arteries ligated as well as the abdominal aorta proximal to the superior mesenteric artery. Retrograde aortic perfusion of the mesenteric arteries and intestine was then performed with 30 mL of cold, phosphate buffered saline followed by 10 mL of an RNA stabilization reagent (RNA*later*; Ambion, Austin, TX).

For immunostaining experiments, control and collateral arteries were harvested at 1–3 days postligation after perfusion with warm phosphate-buffered saline containing a dilator cocktail (0.1 mmol/L adenosine and 0.01 mmol/L sodium nitroprusside) followed by 10% neutral buffered zinc formalin containing the same dilators. Some SHR were treated with the antioxidant apocynin (3 mmol/L in drinking water) beginning 1 day before model creation and continuing until perfusion fixation at 3 days postligation. This dose restores both a normal redox status and collateral growth capacity in SHR mesenteric arteries (Miller et al. 2007a; Zhou et al. 2008).

In experiments to assess the importance of the angiotensin type 1 receptor (AGTR1) in successful collateral luminal expansion, luminal diameters were imaged with maximal dilation at the time of model creation and then 7 days later in rats with and without losartan pretreatment as previously described (Unthank et al. 1996a,b; Miller et al. 2007a).

To assess the potential role of NFkB in collateral growth, experiments were performed in which p65 or control siRNA were administered via tail vein using the hydrodynamic method as previously described (Modlinger et al. 2006; Palm et al. 2007). Briefly, p65 siRNA (ON-Target Plus SmartPool duplex, Thermo Scientific/Dharmacon, Pittsburgh, PA) or nonsense control siRNA (Thermo/Dharmacon; SmartPool universal siRNA control) was reconstituted with RNase-free water and diluted with Mirus Bio TransIT in vivo transfection agent. The siRNA was injected as a 6 mL bolus containing 50 and 25 μg, respectively, at 1 day prior to and 4 days after collateral model creation and luminal expansion assessed by diameter measurements as described above.

### Global gene expression analysis

**Figure 1:**
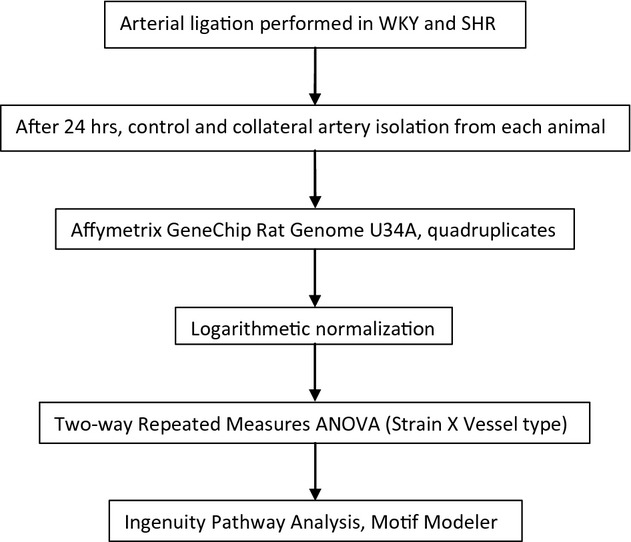
Experimental design for microarray analysis. Microarray analysis utilizing the Affymetrix GeneChip Rat Genome U34A Array was performed on control and collateral mesenteric arteries of WKY and SHR (N = 4 each). Data were extracted using Affymetrix Microarray Suite 5.0 software and statistical analysis for a within subject design was performed after logarithmetic normalization. Data mining was then performed with IPA to identify molecules with the greatest expression changes, top biological functions, and canonical pathways. Motif Modeler was used to find predicted transcription factor binding sites in molecules with altered expression in collateral relative to control arteries.

Microarray analysis of WKY and SHR mesenteric arteries was performed by the Indiana University Center for Medical Genomics (IUCMG) using the Affymetrix GeneChip Rat Genome U34A Array as previously described (Miller et al. 2007b) but with amplification (McClintick et al. 2003). Control and collateral arteries from each animal were labeled individually and hybridized to an array. No pooling was used. Data were analyzed using Affymetrix Microarray Suite 5.0 software (MAS5; Affymetrix MicroArray Suite 5.0 User's Guide, Santa Clara, CA). The experimental design for the microarray analysis is summarized in Figure 1. Same-animal control and collateral arteries were harvested from four WKY and four SHR. One WKY control sample had insufficient RNA and was excluded from analysis. A complete data set is available at the NCBI GEO database (http://www.ncbi.nlm.nih.gov/projects/geo) under accession GSE19524. Microarray data analyses were carried out using the Microarray Data Portal, a proprietary analytical and informatics algorithm developed by the IUCMG, and IPA (Ingenuity® Systems, http://www.ingenuity.com). To eliminate data from probe sets that are not reliably detected, only those probe sets identified as “present” by MAS5 in at least half of the arrays were analyzed (McClintick et al. 2003).

### Real-time quantitative RT-PCR (qRT-PCR)

Total RNA was obtained from additional sets of animals and mesenteric artery mRNA expression assessed using real-time quantitative PCR as previously described in detail (Miller et al. 2007b). Aliquots (5.0 μL of 1:5–1:50 dilutions) of reverse transcription reactions (0.5 μg total RNA) were combined with the appropriate primers for targets or the beta-actin endogenous control (TaqMan® Gene Expression Assays; Applied Biosystems, Foster City, CA) in the presence of PCR reagents (QuantiTect™ Probe PCR Kit; Qiagen, Valencia, CA). Reactions were run in triplicate on an Applied Biosystems 7500 Real-Time PCR System using relative quantification (ddCt) with dual-labeled (FAM/MGB) probes as the product detection method. Standard cycling conditions (2-step PCR, 40 cycles) were used for all targets except AGTR1b (angiotensin II receptor, type 1b), which was modified to 45 cycles. Differences in PCR product yields between groups were determined by comparing the fold differences between target mRNA after normalization to beta-actin.

### Immunohistochemistry

Immunostaining was performed using an immunoperoxidase technique on cross-sections of paraffin-embedded control and collateral vessels as previously described (Miller et al. 2007b). Briefly, for immunoperoxidase studies, slides from paraffin blocks were antigen retrieved using DAKO Target Retrieval solution (pH 6.0) to expose antigens masked by formalin. Endogenous biotin was blocked with avidin/biotin blocking system (DAKO) and endogenous peroxidase with 3% hydrogen peroxide. Antibodies were applied for 60 min at room temperature at a concentration determined by titering to eliminate nonspecific background staining. Slides were developed using DAKO's EnVision+ Dual Link (DAKO North America Inc., Carpinteria, CA), HRP kit for rabbit or mouse primary antibodies in a DAKO Autostainer. The AGTR1 polyclonal primary antibody was generated from a peptide immunogen for the sequence LQLLKYIPPKAKSHSNLSTKMSTLSYRPSDNVSSSTKKP (Sigma Prestige Antibody HPA003596). The antibody for NFĸB (nuclear factor kappa light-chain enhancer of activated B cells) was a mouse monoclonal against NFĸB p65 (Santa Cruz, sc-8008). Quantitation was performed by CAL in a blinded manner by determining the percent of cells or nuclei with immunoreactivity within each wall layer (intima, media, and adventitia).

### Statistical and ontological analyses

Two-way repeated measures analysis of variance (strain × vessel type) was used to assess statistical differences in the microarray experiments. For the microarray analysis, false discovery rates were calculated as previously described (Storey and Tibshirani 2003). Genes that had significant expression changes at *P* ≤ 0.05 using log transformed signal values and fold changes ≥1.25 were further analyzed with IPA. By associating these molecules with biological functions in the Ingenuity Knowledge Base, IPA functional analysis identified the biological functions most significant to the data set. Right-tailed Fisher's exact test was used to calculate a *P*-value determining the probability that each biological function was due to chance alone. IPA canonical pathways analysis identified the pathways from the IPA library of canonical pathways that were most significant to the data set. Fisher's exact test was used to calculate a P-value determining the probability that the association between the genes in the data set and the canonical pathway was explained by chance alone. Statistical significance of arterial diameter change and immunoreactivity results was assessed by two-way repeated measures analysis of variance with pairwise multiple comparison procedures performed with the Holm–Sidak method (Systat Software Inc., Sigmaplot for Windows v.11).

## Results

### Differences between WKY and SHR collaterals

**Figure 2:**
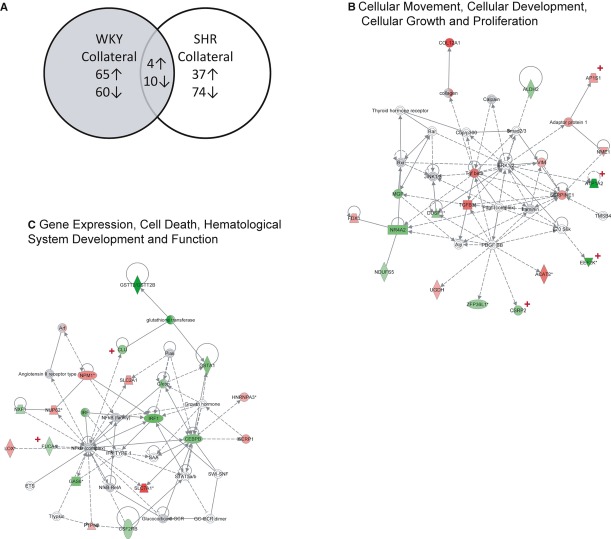
Fundamental differences in collateral gene expression. (A) Venn diagram of number of genes with increased (↑) or decreased (↓) expression in collateral artery relative to same animal control artery for WKY and SHR (*P* ≤ 0.05, fold change ≥1.25×). An IPA comparison analysis of the molecules with altered expression (125 in WKY, 111 in SHR) indicates 222 genes unique to either set, with only 14 common to both sets and demonstrating similar expression changes. The upregulated genes with common expression between WKY and SHR included AP1S1 (adaptor-related protein complex 1, sigma 1 subunit), C6orf115 (ABRA C-terminal like), LRRC59 (leucine rich repeat containing 59), and PTPN2 (protein tyrosine phosphatase, nonreceptor type 2); those downregulated were ATP1A2 (ATPase, Na+/K+ transporting, alpha 2 polypeptide), COX8A (cytochrome c oxidase subunit VIIIA (ubiquitous)), CST3 (cystatin C), ECH1 (enoyl CoA hydratase 1, peroxisomal), FUCA1 (fucosidase, alpha-L- 1, tissue), IDH2 (isocitrate dehydrogenase 2 (NADP+), mitochondrial), LDHB (lactate dehydrogenase B), PPA2 (pyrophosphatase (inorganic) 2), PPP1R1A (protein phosphatase 1, regulatory (inhibitor) subunit 1A), and ZFP36L1(zinc finger protein 36, C3H type-like 1). The two IPA highest scored networks in WKY were Cellular Movement, Cellular Development, Cellular Growth and Proliferation shown in (B), and Gene Expression, Cell Death, Hematological System Development and Function depicted in (C). Molecules with significant up- and downregulation are identified with red and green shading, respectively. Comparison of these molecules between WKY and SHR demonstrated fundamental differences. For those genes in the Cellular Movement, Cellular Development, Cellular Growth and Proliferation, only AP1S1(adaptor-related protein complex 1, sigma 1 subunit), ATP1A2, EEF2K (eukaryotic elongation factor-2 kinase), and CSRP2 (cysteine and glycine-rich protein 2) had altered expression in SHR (red +). Similarly, within the Gene Expression, Cell Death, Hematological System Development and Function network, only CLU (clusterin) and FUCA (fucosidase, alpha-L- 1, tissue) had altered collateral expression in SHR (red +). □ = cytokine, ⋄ = enzyme, ○ = other, horizontal oval = transcription regulator, ▲= phosphatase, ▼ = kinase, vertical oval = transmembrane receptor, trapezoid = transporter. Lines without arrows indicate binding, lines with arrows indicate stimulation, solid lines indicate direct interaction, and dashed lines indirect.

A primary goal of the study was to identify potential molecules and discover related functions and pathways that might mediate the collateral growth impairment observed in SHR. To accomplish this objective in an unbiased manner, analysis of the microarray data was performed with IPA. The first objective was to determine the total number of molecules with altered expression between collateral and control arteries in SHR and WKY. The number of genes present on the microarray with adequate expression for analysis was 1795. IPA was used to determine the molecules with altered expression in collateral compared to control arteries (within subject design, *P* ≤ 0.05, fold change ≥1.25×) that were both common and unique to WKY and SHR. There were a total of 125 and 111 genes with altered expression in WKY and SHR, respectively. Figure 2 reports the number of these molecules which were up- and downregulated, and shows that only 14 were common between WKY and SHR. These results indicate a fundamental difference between WKY and SHR in the global pattern of collateral gene expression. The potential impact of this difference between WKY and SHR was further assessed in IPA by analyses of networks, biological functions, and canonical pathways.

#### Network analyses

The five gene networks with the highest scores determined by IPA are shown in Table 1 for WKY and SHR. In WKY, the networks with the two highest scores were (1) Cellular Movement, Cellular Development, Cellular Growth and Proliferation, and (2) Gene Expression, Cell Death, and Hematological System Development and Function. We examined these two networks to further assess collateral expression differences between these two strains. As illustrated in Figure 2B and C, these two networks contained 18 and 17 molecules, respectively, with altered expression (*P* ≤ 0.5 and fold change ≥1.25×) in WKY collaterals. Comparison to the molecules altered in the SHR collaterals for these two WKY networks revealed that only six molecules combined were common between strains (molecules identified by red plus signs in Figure 2B and C).

**Table 1:** IPA highest ranked networks

Network ID	Associated network functions	Score
(A) WKY		
1	Cellular Movement, Cellular Development, Cellular Growth and Proliferation	34
2	Gene Expression, Cell Death, Hematological System Development and Function	31
3	Gene Expression, Cancer, Small Molecule Biochemistry	29
4	Lipid Metabolism, Small Molecule Biochemistry, Vitamin and Mineral Metabolism	26
5	Gene Expression, Cell Death, Gastrointestinal Disease	25
(B) SHR		
1	Cell-to-Cell Signaling and Interaction, Nervous System Development and Function, Cell Cycle	42
2	Cellular Movement, Cell Cycle, Cellular Growth and Proliferation	37
3	Cellular Development, Hepatic System Development and Function, Lipid Metabolism	30
4	Cellular Assembly and Organization, Cell Death, DNA Replication, Recombination, and Repair	29
5	Genetic Disorder, Neurological Disease, Skeletal and Muscular Disorders	25

IPA, Ingenuity® pathway analysis; WKY, Wistar Kyoto; SHR, spontaneously hypertensive rat.

#### Biological functions

Table 2 lists the five highest IPA rated biological functions within the molecular and cellular category for collateral artery gene expression in WKY and SHR. Not only are the top functions dissimilar for the WKY and SHR, but evaluation of the specific molecules altered within WKY and SHR collaterals display limited overlap or similarity for a given function. For example, of altered molecules in the functional groups of Cellular Growth and Proliferation and Cell Cycle (among top ranked for WKY and SHR, respectively, Table 2), only 1 molecule was common (PTPN2). In addition, Gene Expression, although not among the IPA highest ranked Bio Functions for either WKY or SHR, was included in the list of significantly altered molecular and cellular functions for both and included 39 molecules in WKY (39 subcategories with *P*-values from 3.43 × 10^−6^ to 2.09 × 10^−3^) and eight in SHR (eight subcategories, *P* = 3.40 × 10^−5^ to 1.27 × 10^−2^) without a single molecule in common.

**Table 2:** IPA highest ranked molecular and cellular functions

Name	*P*-value	# Molecules
(A) WKY Collateral vs. Control Arteries		
Cellular Development	1.96E-08–1.75E-03	50
Cellular Growth and Proliferation	1.96E-08–2.06E-03	54
Cell-To-Cell Signaling and Interaction	6.39E-08–2.06E-03	40
Cell Death	1.32E-07–2.09E-03	49
Cellular Movement	1.84E-07–2.09E-03	37
(B) SHR Collateral vs. Control Arteries		
Cellular Assembly and Organization	2.07E-05–1.27E-02	19
Free Radical Scavenging	2.41E-05–8.06E-03	12
Molecular Transport	2.41E-05–1.27E-02	35
Cell Cycle	2.49E-05–1.27E-02	22
Protein Synthesis	2.71E-05–8.33E-04	14

IPA, Ingenuity® pathway analysis; WKY, Wistar Kyoto; SHR, spontaneously hypertensive rat.

#### Canonical pathway analysis

**Figure 3:**
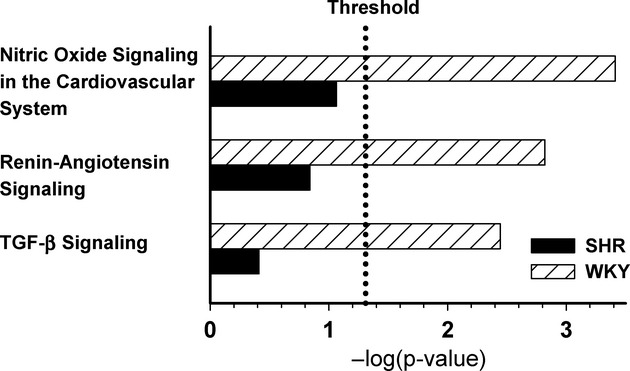
Canonical pathways within IPA known to the authors to have important roles in various types of arterial remodeling and which exhibited significant alterations in WKY but not SHR included signaling pathways associated with nitric oxide, the renin–angiotensin system (RAS), and transforming growth factor-beta as shown in this figure.

Based upon the functional analyses, canonical pathways within IPA were selected for the categories of Cardiovascular Signaling; Cell Cycle Regulation; Cellular Growth, Proliferation, and Development; and Transcriptional Regulation and further screened based upon molecular ratio ≥0.03 and statistical significance at *P* ≤ 0.01. The canonical pathways which met these criteria and were different between WKY and SHR are reported in Table 3. Significant alterations in WKY but not SHR were observed for signaling pathways associated with nitric oxide, the renin–angiotensin system (RAS), and transforming growth factor-beta (Fig. 3), all of which are known to have important roles in various types of arterial remodeling. In SHR, the canonical pathways of mitochondrial dysfunction and insulin receptor signaling were altered.

**Table 3:** IPA highest ranked canonical pathways

Ingenuity canonical pathways	Analysis name	Ratio	*P*-value	Molecules
(A) Significantly altered in WKY but not SHR				
Nitric Oxide Signaling in the Cardiovascular System	WKY	0.050	0.000	PRKAR2B,PLN,GUCY1A3,SLC7A1,ATP2A3
Nitric Oxide Signaling in the Cardiovascular System	SHR	0.020	0.091	ITPR3,ATP2A2
IL-1 Signaling	WKY	0.047	0.001	GNB1,FOS,JUN,PRKAR2B,ADCY6
IL-1 Signaling	SHR	0.019	0.117	GNG11,GNB2
TGF-β Signaling	WKY	0.045	0.003	FOS,JUN,TGFB3,SERPINE1
TGF-β Signaling	SHR	NA	NA	
Renin–Angiotensin Signaling	WKY	0.040	0.001	FOS,JUN,PRKAR2B,ADCY6,AGT
Renin–Angiotensin Signaling	SHR	0.016	0.154	ITPR3,AGTR1
(B) Significantly altered in SHR but not WKY				
Mitochondrial Dysfunction	SHR	0.040	0.000	NDUFS7,XDH,COX8A,COX7A2L,APP,NDUFB2,NDUFB10
Mitochondrial Dysfunction	WKY	0.017	0.079	NDUFS5,COX8A,TXNRD2
Insulin Receptor Signaling	SHR	0.036	0.001	RPS6KB1,BAD,EIF2B3,GSK3B,PPP1CA
Insulin Receptor Signaling	WKY	0.014	0.254	PRKAR2B,EIF4EBP1

ADCY6, adenylate cyclase 6; AGT, angiotensinogen; AGTR1, angiotensin II receptor, type 1; APP, amyloid beta (A4) precursor protein; ATP2A2, ATPase, Ca++ transporting, cardiac muscle, slow twitch 2; ATP2A3, ATPase, Ca++ transporting, ubiquitous; BAD, BCL2-associated agonist of cell death; COX7A2L, cytochrome c oxidase subunit VIIa polypeptide 2 like; COX8A, cytochrome c oxidase subunit VIIIa; EIF2B3, eukaryotic translation initiation factor 2B, subunit 3; EIF4EBP1, eukaryotic translation initiation factor 4E-binding protein 1; FOS, FBJ osteosarcoma oncogene; GNB1, guanine nucleotide-binding protein (G protein), beta polypeptide 1; GNB2, guanine nucleotide-binding protein (G protein), beta polypeptide 2; GNG11, guanine nucleotide-binding protein (G protein), gamma 11; GSK3B, glycogen synthase kinase 3 beta; GUCY1A3, guanylate cyclase 1, soluble, alpha 3; ITPR3, inositol 1,4,5-trisphosphate receptor, type 3; JUN, jun proto-oncogene; NDUFB10, NADH dehydrogenase (ubiquinone) 1 beta subcomplex, 10; NDUFB2, NADH dehydrogenase (ubiquinone) 1 beta subcomplex, 2; NDUFS5, NADH dehydrogenase (ubiquinone) Fe-S protein 5; NDUFS7, NADH dehydrogenase (ubiquinone) Fe-S protein 7; PLN, phospholamban; PPP1CA, protein phosphatase 1, catalytic subunit, alpha isozyme; PRKAR2B, protein kinase, cAMP dependent regulatory, type II beta; PRKAR2B, protein kinase, cAMP dependent regulatory, type II beta; PRKAR2B, protein kinase, cAMP dependent regulatory, type II beta; RPS6KB1, ribosomal protein S6 kinase, polypeptide 1; SERPINE1, serpin peptidase inhibitor, clade E (nexin, plasminogen activator inhibitor type 1), member 1; SLC7A1, solute carrier family 7 (cationic amino acid transporter, y+ system), member 1; TGFB3, transforming growth factor, beta 3; TXNRD2, thioredoxin reductase 2; XDH, xanthine dehydrogenase. IPA, Ingenuity® pathway analysis; WKY, Wistar Kyoto; SHR, spontaneously hypertensive rat; IL, interleukin; TGF, transforming growth factor.

#### Molecules with greatest fold changes

Comparison of the molecules with the greatest fold changes were identified by IPA and further confirmed fundamental differences in gene expression between WKY and SHR collaterals. Table 4 reports the top ten up- and downregulated molecules in WKY and also shows the corresponding expression change, if any, in the SHR. Only one of the top 10 up- and downregulated molecules in WKY collaterals had significantly altered expression in the SHR (ATP1A2, ATPase, Na+/K+ transporting, alpha 2 polypeptide). Additional molecules upregulated in the WKY but not SHR included L-arginine transporter solute carrier family 7 (SLC7A1), interleukin 1β (IL1β), and transforming growth factor β3 (TGFβ3); while angiotensinogen (AGT), jun proto-oncogene (JUN), and neurotactin or fractalkine (CX3CL1) were among the molecules downregulated only in WKY collaterals. Similar comparison of the molecules with the greatest fold changes in SHR (Table 5) revealed that none of the top 10 upregulated molecules were altered in WKY while only three of the greatest downregulated molecules exhibited decreased expression in WKY collaterals (ECH1, enoyl CoA hydratase 1, peroxisomal; ATP1A2; CST3, cystatin C). Growth arrest and DNA-damage-inducible α (GADD45α) was among the top 10 genes upregulated in SHR but not WKY collaterals.

**Table 4:** Molecules with greatest fold changes in collateral expression in WKY with comparison to SHR

WKY fold change	WKY *P*-value	SHR fold change	SHR *P*-value	Symbol	Entrez gene name
3.97	0.002	1.72	0.413	ESM1	Endothelial cell-specific molecule 1
2.84	0.012	−1.00	0.989	COL12A1	Collagen, type XII, alpha 1
2.65	0.010	1.22	0.505	SLC7A1	Solute carrier family 7 (cationic amino acid transporter, y+ system), member 1
2.48	0.019	1.41	0.422	IL1B	Interleukin 1, beta
2.42	0.035	1.70	0.174	DIO3	Deiodinase, iodothyronine, type III
2.37	0.010	−0.98	0.925	ACTA1	Actin, alpha 1, skeletal muscle
2.28	0.031	−1.01	0.984	CRYAB	Crystallin, alpha B
2.25	0.002	−1.05	0.899	IDI1	Isopentenyl-diphosphate delta-isomerase 1
2.25	0.007	−1.79	0.123	TGFB3	Transforming growth factor, beta 3
2.19	0.041	1.31	0.419	CYP51A1	Cytochrome P450, family 51, subfamily A, polypeptide 1
					
−6.45	0.039	−1.27	0.595	AGT	Angiotensinogen (serpin peptidase inhibitor, clade A, member 8)
−4.08	0.016	−2.61	0.053	SULT1A1	Sulfotransferase family, cytosolic, 1A, phenol-preferring, member 1
−3.44	0.048	−1.32	0.243	TNXA	Tenascin XA
−2.93	0.049	−2.08	0.679	JUN	Jun proto-oncogene
−2.86	0.000	−1.97	0.128	GSTT2	Glutathione S-transferase theta 2
−2.84	0.010	−2.21	0.034	ATP1A2	ATPase, Na+/K+ transporting, alpha 2 polypeptide
−2.74	0.044	−1.65	0.113	EPHX1	Epoxide hydrolase 1, microsomal (xenobiotic)
−2.68	0.001	−2.32	0.623	FMOD	Fibromodulin
−2.49	0.029	−1.86	0.182	EEF2K	Eukaryotic elongation factor-2 kinase
−2.48	0.025	−0.68	0.831	CX3CL1	Chemokine (C-X3-C motif) ligand 1

IPA, Ingenuity® pathway analysis; WKY, Wistar Kyoto; SHR, spontaneously hypertensive rat.

**Table 5:** Molecules with greatest fold changes in collateral expression in SHR with comparison to WKY

SHR fold change	SHR*P*-value	WKY fold change	WKY *P*-value	Symbol	Entrez gene name
3.40	0.049	1.38	0.822	CYP4A14	Cytochrome P450, family 4, subfamily a, polypeptide 14
1.91	0.011	1.10	0.650	AKAP11	A kinase (PRKA) anchor protein 11
1.91	0.034	1.16	0.773	GADD45A	Growth arrest and DNA-damage-inducible, alpha
1.89	0.047	1.72	0.172	EIF2B3	Eukaryotic translation initiation factor 2B, subunit 3 gamma, 58 kDa
1.80	0.048	−1.14	0.773	NAPA	N-ethylmaleimide-sensitive factor attachment protein, alpha
1.73	0.030	1.57	0.164	LOC686240	Similar to NMDA receptor regulated 1-like
1.72	0.015	1.05	0.833	MRPL24	Mitochondrial ribosomal protein L24
1.71	0.018	−0.95	0.888	ACSL4	Acyl-CoA synthetase long-chain family member 4
1.68	0.037	1.05	0.876	GSK3B	Glycogen synthase kinase 3 beta
1.68	0.018	−1.03	0.878	GFM1	G elongation factor, mitochondrial 1
−2.93	0.006	−1.58	0.638	THY1	Thy-1 cell surface antigen
−2.41	0.017	−2.23	0.018	ECH1	Enoyl CoA hydratase 1, peroxisomal
−2.31	0.015	−1.11	0.750	HSD3B7	Hydroxy-delta-5-steroid dehydrogenase, 3 beta- and steroid delta-isomerase 7
−2.21	0.034	−2.84	0.010	ATP1A2	ATPase, Na+/K+ transporting, alpha 2 polypeptide
−2.06	0.047	−1.14	0.658	IRF3	Interferon regulatory factor 3
−1.99	0.006	−1.07	0.768	PTPRA	Protein tyrosine phosphatase, receptor type, A
−1.88	0.010	1.09	0.679	SLC6A6	Solute carrier family 6 (neurotransmitter transporter, taurine), member 6
−1.86	0.005	−1.13	0.214	ITGA7	Integrin, alpha 7
−1.83	0.038	−1.98	0.000	CST3	Cystatin C
−1.83	0.015	−1.54	0.055	IVD	Isovaleryl-CoA dehydrogenase

IPA, Ingenuity® pathway analysis; WKY, Wistar Kyoto; SHR, spontaneously hypertensive rat.

### Differences between WKY and SHR control arteries

Next, comparisons between SHR and WKY control arteries were made to determine if additional insight might be obtained to explain the fundamental difference in collateral gene expression between these two rat strains. In SHR control arteries, 175 molecules had altered expression relative to WKY controls (≥1.25 fold change, *P* ≤ 0.05). The IPA highest rated networks, cellular and molecular biological functions, and molecules with greatest fold changes are reported in Table 6. Cell growth, cell cycle, cell proliferation, and gene expression were biological processes included within the highest rated gene networks and molecular and cellular functions (Table 6A and B). Molecules with the greatest upregulation in SHR relative to WKY were CD74 (HLA class II histocompatibility antigen gamma chain) and HLA-DR (IK cytokine, downregulator of HLA II) (Table 6C). CX3CL1 was the molecule with the greatest downregulation. Six of the 10 molecules with the greatest degree of downregulation in SHR control arteries were molecules involved in transcriptional regulation, including JUN, EGR1 (early growth response 1), and EGR2 (early growth response 2). In addition to these molecules with the greatest fold changes, several other molecules of potential significance in terms of impacting flow-mediated outward remodeling also exhibited altered expression. As can be seen in the online database, these include CYBA (cytochrome b-245, alpha polypeptide or p22phox), an NAD(P)H oxidase component, and NFĸB1 (nuclear factor of kappa light polypeptide gene enhancer in B cells 1) which gives rise to p105 and the DNA-binding component of NFĸB, p50. CYBA expression was increased 3.2× (*P* = 0.027) in SHR; NFĸB1 was downregulated 1.95× (*P* = 0.044).

**Table 6:** Summary of IPA analysis for SHR versus WKY control arteries

(A) Highest ranked networks		
Network ID	Associated network functions	Score
1	Cell Death, Gene Expression, Cellular Growth and Proliferation	35
2	Antigen Presentation, Cell-To-Cell Signaling and Interaction, Hematological System Development and Function	29
3	Inflammatory Response, Cell Death, Cell-To-Cell Signaling and Interaction	26
4	PostTranslational Modification, Protein Folding, Protein Degradation	26
5	Protein Synthesis, Cell Cycle, RNA PostTranscriptional Modification	24

(B) Top biological functions: molecular and cellular functions		
Name	*P*-value	# Molecules
Cell Cycle	2.29E-06–2.05E-02	19
Cell Death	2.29E-06–1.88E-02	30
Cellular Development	2.29E-06–1.99E-02	26
Gene Expression	1.90E-05–2.11E-02	28
Cellular Growth and Proliferation	4.75E-05–1.99E-02	34

(C) Molecules with greatest fold changes in SHR vs. WKY control arteries			
Fold change	*P*-value	Symbol	Entrez gene name
12.34	0.006	CD74	CD74 molecule, major histocompatibility complex, class II invariant chain
12.27	0.002	HLA-DRA	IK cytokine, downregulator of HLA II
12.15	0.033	APOD	Apolipoprotein D
5.94	0.004	FCGR2A	Fc fragment of IgG, low affinity IIa, receptor (CD32)
5.36	0.003	MAF	V-maf musculoaponeurotic fibrosarcoma oncogene homolog (avian)
5.33	0.007	THBS4	Thrombospondin 4
4.94	0.028	DMBT1	Deleted in malignant brain tumors 1
4.77	0.002	ENTPD2	Ectonucleoside triphosphate diphosphohydrolase 2
4.45	0.012	FMO1	Flavin-containing monooxygenase 1
4.45	0.029	LYZ	Lysozyme
−11.24	0.031	CX3CL1	Chemokine (C-X3-C motif) ligand 1
−9.87	0.009	NR4A3	Nuclear receptor subfamily 4, group A, member 3
−6.59	0.007	VCAM1	Vascular cell adhesion molecule 1
−5.47	0.002	KLF6	Kruppel-like factor 6
−5.37	0.024	DRD1	Dopamine receptor D1
−5.17	0.023	JUN	Jun proto-oncogene
−4.08	0.001	IRF1	Interferon regulatory factor 1
−4.05	0.012	EGR1	Early growth response 1
−3.97	0.001	DUSP1	Dual specificity phosphatase 1
−3.70	0.002	EGR2	Early growth response 2

IPA, Ingenuity® pathway analysis; WKY, Wistar Kyoto; SHR, spontaneously hypertensive rat.

### Transcription factor binding sites

The IPA analyses described above implicated potential differences in transcription factor expression and transcriptional regulation between SHR and WKY in collateral and control arteries. Therefore, Motif Modeler (Liu et al. 2006) was used to identify predicted transcription factor binding sites based on the molecules whose gene expression was altered in the collateral arteries. Potential binding sites were found for 19 potential transcription factors in WKY and SHR. Remarkably, there was no overlap in the predicted transcription factors between WKY and SHR (Table 7). Several of the identified binding sites in WKY collaterals were for transcription factors known to be sensitive to mechanical stimulation (shear stress and/or circumferential wall stress) or redox status, including at least three known to be influenced by both mechanical stimuli and reactive oxygen or nitrogen species (NFĸB [Allen and Tresini 2000; Davis et al. 2004; Lan et al. 1994], AP-1[activator protein 1] [Lan et al. 1994; Allen and Tresini 2000], and NF-E2 [nuclear factor, erythroid derived 2] [Dai et al. 2007; Warabi et al. 2007]).

**Table 7:** Number of altered genes with predicted transcription factor binding sites in WKY and SHR

(A) Altered in WKY			(B) Altered in SHR		
	
Transcription factor	WKY #	SHR #	Transcription factor	WKY #	SHR #
GATA-1	24	0	AML-1a	0	54
Zic1	24	0	AML1	0	54
PR	23	0	Pax-2	0	24
NF-E2	22	0	IRF-1	0	20
AP-1	22	0	MyoD	0	19
STAT3	22	0	PPARα:RXR-α	0	19
NF-kappaB (p65)	21	0	NERF1a	0	18
Ik-1	20	0	Myogenin	0	18
Zic2	20	0	E2A	0	18
GR	20	0	TATA	0	17
PBX	19	0	E12	0	17
YY1	18	0	SMAD-4	0	17
CDP	13	0	PEBP	0	15
SOX-9	13	0	SF-1	0	14
Crx	13	0	T3R	0	14
ELF-1	13	0	ERR alpha	0	13
HNF4, COUP	12	0	MEIS1A:HOXA9	0	12
SREBP-1	11	0	CDP CR1	0	11
OCT1	7	0	Zic3	0	11

Symbol of transcription factor or regulatory-binding site followed by full name and the TFBS accession number in brackets. AML1,acute myeloid leukemia 1 (also known as runt-related transcription factor 1, Runx1) [M00751]; AML-1a, acute myeloid leukemia 1a, transcription factor encoded by AML1 [M00271]; AP-1, activator protein 1 [M00174, M00188, M00925, M00926]; CDP, CCAAT displacement protein/Cut homeobox [M00095]; CDP CR1, Cut repeat 1 of CDP [M00104]; Crx, cone-rod homeobox [M00623]; E12, E box protein E12 [M00693], E2A, E2A immunoglobulin enhancer-binding factors E12/E47 (also known as TCF3, transcription factor 3 [M00973], ELF-1, E74-Like Factor 1 (Ets Domain Transcription Factor) [M00746]; ERR alpha, estrogen-related receptor, alpha [M00511]; GATA-1, GATA-binding protein 1 [M00127]; GR, Glucocorticoid receptor (also known as nuclear receptor subfamily 3, group C, member 1, NR3c1) [M00955]; HNF4, hepatocyte nuclear factor 4, alpha [M00967]; Ik-1, IKZF1-IKAROS family zinc finger 1 [M00086]; IRF-1, interferon regulatory factor 1 [M00062]; MEIS1A:HOXA9, homeobox A9 [M00420]; MyoD, myogenic differentiation 1 [M00001, M00184]; myogenin [M00712]; NF-E2, nuclear factor, erythroid derived 2 [M00037]; NERF1a, E74-like factor 2 (ets domain transcription factor) [M00531]; NF-kappaB (p65), nuclear factor of kappa light polypeptide gene enhancer in B cells [M00052]; OCT1, organic cation transporter 1, POU class 2 homeobox 1 [M00135]; Pax-2, paired box 2 [M00486]; PBX, pre-B-cell leukemia homeobox 1 [M00998]; PEBP, phosphatidylethanolamine-binding protein 1 [M00984]; PPARα, peroxisome proliferator-activated receptor alpha [M00518]; SF-1, splicing factor 1 [M00727]; SMAD-4, SMAD family member 4 [M00733]; SOX-9, SRY (sex determining region Y)-box 9 [M00410]; SREBP-1, Sterol regulatory element-binding transcription factor 1 [M00220]; T3R, Thyroid hormone receptors [M00963]; TATA, TATA box [M00216]; YY1, YY1 transcription factor [M00059]; Zic1, Zic family member 1 [M00448]; Zic2, Zic family member 2 [M00449]; Zic3, Zic family member 3 [M00450]. WKY, Wistar Kyoto; SHR, spontaneously hypertensive rat.

### Assessment of the potential functional significance of microarray results

**Figure 4:**
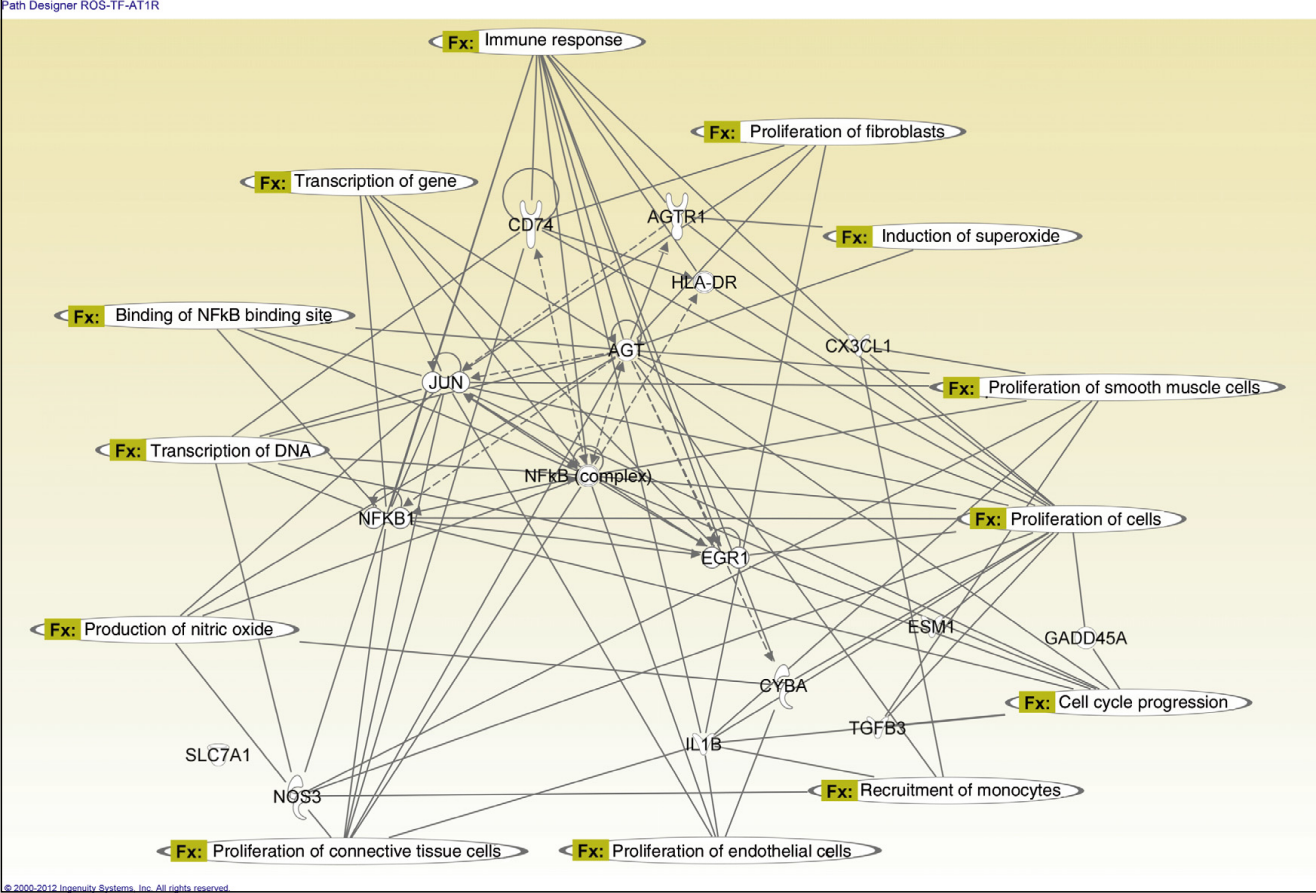
IPA constructed network of key molecules selected from those with the greatest fold changes (Esm1, CX3CL1, CD74, HLA-DR, TGFβ3, ILβ1, GADD45α, SLC7A1), members of highest scored Canonical pathways (eNOS, SLC7A1, AGT, AT1R, TGFβ3), and the NAD(P)H oxidase component, CYBA. Altered regulation of several of these molecules was confirmed by RT-PCR (Table 8). IPA generated connections between the molecules and overlaid specific functions.

A pathway list was created in IPA which included molecules selected from those with the greatest fold changes (ESM1, endothelial specific molecule 1; CX3CL1; CD74; HLA-DR; TGFβ3; ILβ1; GADD45α; SLC7A1), members of the highest ranked Canonical pathways (eNOS, endothelial nitric oxide synthase; SLC7A1; AGT, angiotensinogen; AGTR1b, TGFβ3), and the NAD(P)H oxidase component, CYBA. The interconnecting pathway and biological functions generated by IPA from this list are shown in Figure 4. Altered regulation of several of these was confirmed by RT-PCR (Table 8).

**Table 8:** Comparison of microarray and RT-PCR analyses for selected genes important in arterial remodeling

	Microarray		RT-PCR	
		
	WKY	SHR	WKY	SHR
(A) Control artery relative expression				
Cyba	393 ± 167	1104 ± 169†	0.928 ± 0.095	2.140 ± 0.495†
(B) Collateral/control ratio				
Agt	0.159 ± 0.106*	0.674 ± 0.415	0.401 ± 0.088*	0.563 ± 0.288
AGTR1b	0.463 ± 0.208	0.482 ± 0.198*	3.018 ± 0.703*^,^†	0.425 ± 0.156†
CX3CL1	0.564 ± 0.376*	0.645 ± 0.394	0.533 ± 0.102*	0.479 ± 0.062*
ESM1	3.99 ± 1.298*	1.81 ± 0.545	4.758 ± 1.159*	2.169 ± 0.356*

Data are given as relative expression for cyba in control arteries and for collateral/control ratios for the remaining molecules. Results between Mircoarray and RT-PCR were similar except for AGTR1b in WKY where expression was increased rather than decreased, (N ≥ 4; *, **†**, **‡**: P ≤ 0.05 Collateral vs. Control, SHR vs. WKY, qRT-PCR vs. Microarray). Cyba, cytochrome b-245, alpha polypeptide, p22-phox; Agt, angiotensinogen; AGTR1b, angiotensin II receptor, type 1b; CX3CL1, chemokine (C-X3-C motif) ligand 1; ESM1, endothelial cell-specific molecule 1. WKY, Wistar Kyoto; SHR, spontaneously hypertensive rat.

**Figure 5:**
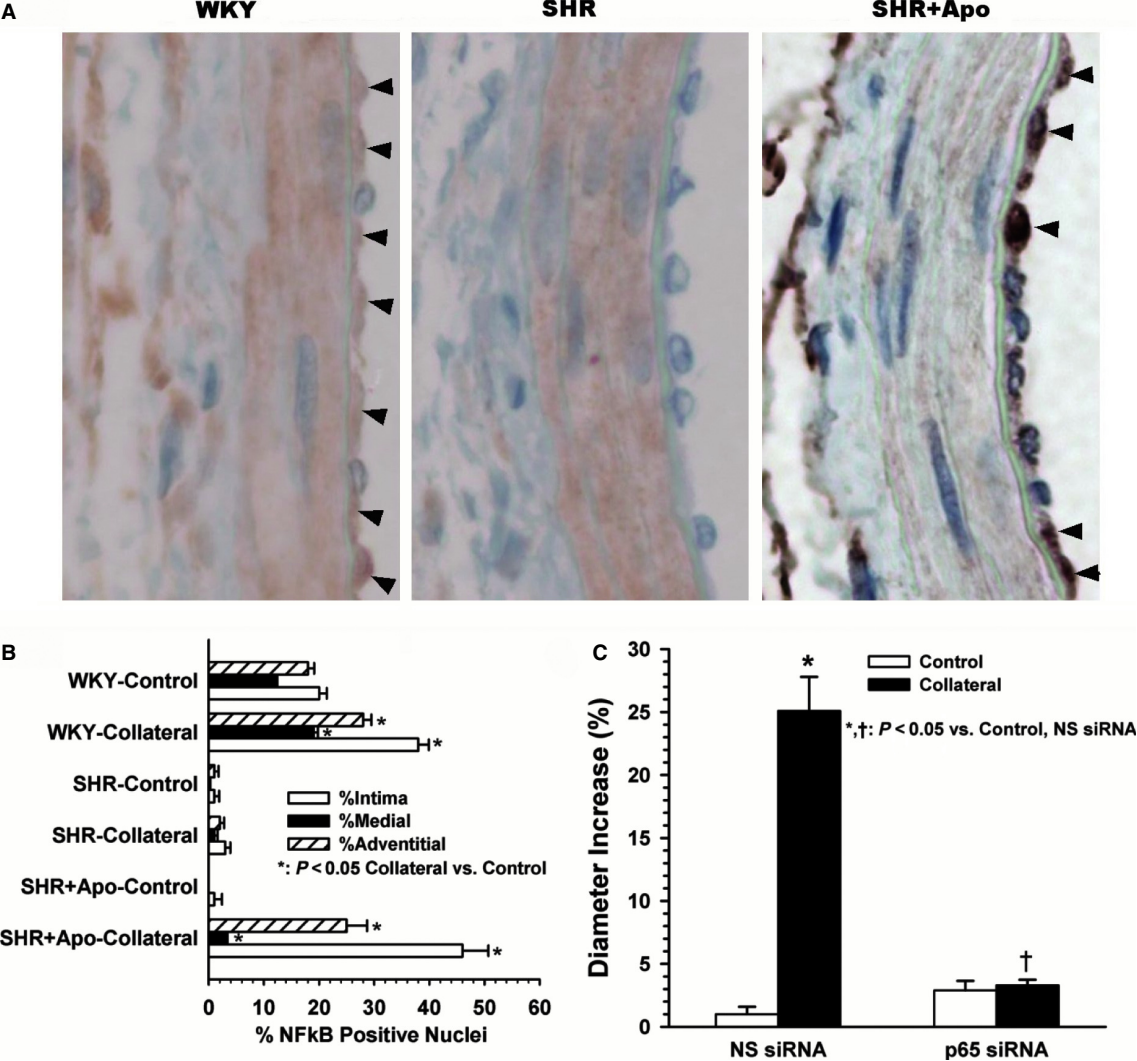
Nuclear localization of NF-κB during successful and impaired collateral growth. (A) Representative images showing NF-κB immunoreactivity (brown) in collateral arteries 3 days after arterial ligation in WKY, SHR, and apocynin pretreated SHR (SHR+Apo). Nuclear localization is apparent especially within the intima of WKY and SHR+Apo, as indicated by arrows, but not in SHR. (B) Analysis of the percentage of cells with immunoreactivity in each wall layer indicates a statistical increase in all wall layers of collaterals from WKY and SHR+Apo relative to same animal controls, (*n* ≥ 3, *P ≤ 0.001). (C) Inhibition of p65 expression suppressed collateral growth. Paired comparisons of control and collateral arteries before and 7 days after arterial ligation demonstrated significant collateral enlargement in WKY administered a control nonsense siRNA (*P ≤ 0.001, *n* = 4) but not in WKY pretreated with siRNA to p65 (^†^*P* ≤ 0.001 vs. nonsense siRNA collateral, *n* = 3). No effect of p65 siRNA was observed on the diameters of control arteries.

NFĸB was central to the generated pathway and linked to the major functions of gene expression, cell growth and proliferation, and cell cycling. It was included within the list of transcription factors with predicted binding sites in genes altered in WKY but not SHR collaterals that were sensitive to both mechanical stimuli and redox status. To assess its potential role, NFĸB immunoreactivity and nuclear localization were assessed in control and collateral arteries in SHR and WKY rats. Representative arterial cross-sections are shown in Figure 5A. While there was a remarkable increase in the percent of nuclei with immunoreactivity in the intima, media, and adventitia of WKY collaterals relative to same animal control arteries, no such increase was observed in the SHR collaterals (Fig. 5B). As changes in redox status have been reported to both activate and inhibit NFĸB (Allen and Tresini 2000; Grumbach et al. 2005), additional SHR rats were treated with the antioxidant apocynin which restores a normal redox status in SHR mesenteric arteries (Zhou et al. 2008). When arterial ligation was performed in these animals, nuclear localization occurred within the collaterals similar to that observed within the WKY rats (Fig. 5A and B). To further assess the role for NFkB in collateral growth, we assessed the effect of inhibiting p65 expression with siRNA. The results demonstrated that WKY receiving p65 siRNA had significantly suppressed collateral growth compared with rats receiving a control (nonsense) siRNA (Fig. 5C).

**Figure 6:**
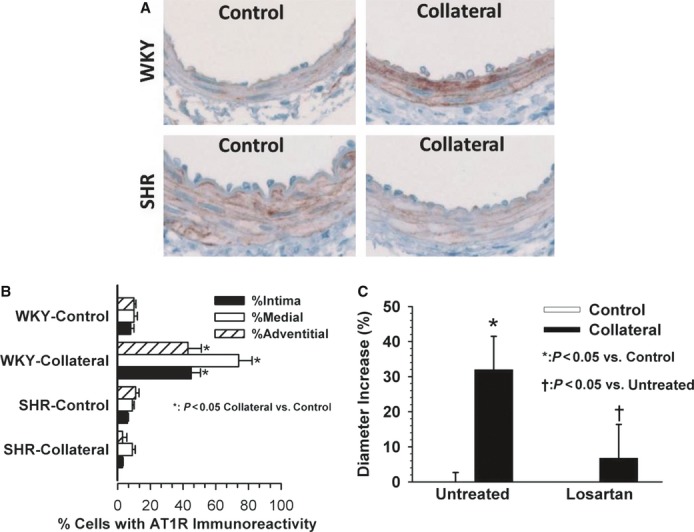
Potential role of the AGTR1 in collateral growth. (A) Representative images showing AGTR1 immunoreactivity (brown) in WKY and SHR control and collateral arteries 3 days after arterial ligation. Relative to same animal controls, a remarkable increase is observed in all wall layers of the WKY but not SHR collateral. (B) Analysis of the percentage of cells with immunoreactivity in each wall layer indicates a statistical increase in all wall layers of the WKY collateral and a decrease in the adventitia of the SHR collateral compared to same animal control artery (*n* = 3, *P ≤ 0.05). C. Effect of AGTR1 blockade by losartan on collateral diameter enlargement in WKY. Paired comparisons of control and collateral arteries before and 7 days after arterial ligation demonstrated significant collateral enlargement in WKY-untreated animals (*P ≤ 0.001) but not in WKY pretreated with losartan (*P* = 0.215), which prevents collateral growth in WKY. No effect of losartan was observed on the diameters of control arteries, consequently the open bars are not apparent on the graph (*n* = 5). The combined data suggest that the upregulation and activation of the AGTR1 is not only correlated with, but is also required for collateral growth in the young normotensive WKY.

One deviation of the RT-PCR results from the microarray analysis was the increased rather than decreased expression of the AGTR1b in WKY. Because of the potentially important role of the RAS and especially AGTR1 in arterial remodeling, additional studies were performed to assess the role of the AGTR1 in collateral growth. Immunostaining for the AGTR1 showed a remarkable increase throughout the arterial wall of WKY but not SHR collaterals (Fig. 6A and B). A functional role for the AGTR1 in successful collateral growth was assessed in additional experiments in which a group of WKY received losartan to suppress AGTR1 activation. These results demonstrated that collateral diameter enlargement was prevented by pretreatment with losartan (Fig. 6C). This observation is consistent with a requirement for increased AGTR1 expression/activation and thus RAS-dependent signaling in successful collateral growth.

## Discussion

To our knowledge, this is the first microarray study to compare primary collateral arteries from animals with and without known cardiovascular risk factors. Comparisons between the normotensive WKY and the SHR (1) demonstrate profound differences in the overall collateral gene expression, (2) suggest redox-dependent transcriptional regulation as a fundamental underlying cause, and (3) identify important biological functions and molecular pathways that may mediate the impaired remodeling of collateral arteries in the presence of cardiovascular risk factors. Advantages and limitations of the model and approach and the potential significance of these observations are considered below.

### Advantages and limitations of the model and approach

In the present study, we focused on differences in gene expression between mesenteric collateral arteries in WKY which exhibit significant growth and in SHR collaterals which do not enlarge (Tuttle et al. 2002b; Miller et al. 2007a). The SHR strain was selected because it has multiple vascular risk factors including essential hypertension, metabolic abnormalities, and abnormal redox status (Okamoto and Aoki 1963; Potenza et al. 2005; Zhou et al. 2008) and is documented to have impaired compensation to arterial occlusion in the peripheral circulation including the mesentery and hindlimb (Nelissen-Vrancken et al. 1992, 1993; Scheidegger et al. 1997; Emanueli et al. 2001, 2002; Tamarat et al. 2002; Tuttle et al. 2002b; Srivastava et al. 2003; Iaccarino et al. 2005; Miller et al. 2007a; You et al. 2008; Matsumura et al. 2009). We chose to use the mesenteric model of collateral growth because it has a simple, well-defined collateral pathway that facilitates identification and isolation of the primary collaterals and controls. In addition, vessels from one rat contain sufficient DNA for microarray analysis with amplification. To our knowledge, these are the model and strain combinations that are best characterized in terms of collateral artery blood flow, wall shear rate, and periarterial NO and H_2_O_2_ concentrations (Unthank et al. 1996a,b; Tuttle et al. 2001, 2002b; Zhou et al. 2008). Blood flow and wall shear rate increase in collaterals of both strains to approximately the same degree (Tuttle et al. 2002b). While both flow-mediated collateral growth and NO production are fully suppressed in the SHR mesenteric arteries, both are completely restored by antioxidants (Tuttle et al. 2002b; Miller et al. 2007a; Zhou et al. 2008).

In contrast, in more complex tissues there are multiple potential collaterals which experience unknown hemodynamic and redox changes and enlarge to different degrees, or even regress (Longland 1953; Herzog et al. 2002; Distasi et al. 2009). In addition, collateral vessels in other organs are embedded within the parenchymal tissue and it can be difficult to both identify and isolate them. While it is possible that responses in mesenteric arteries may not represent completely what occurs in other organs, flow-mediated remodeling is a universal phenomenon within the arterial tree (reviewed by Unthank et al. 2011) and available studies indicate similarities in cellular proliferation, matrix activation, and gene expression and impairment by risk factors associated with oxidative stress (Masuda et al. 1989; Unthank et al. 1996a,b; Miyashiro et al. 1997; Tuttle et al. 2001, 2002b; Sho et al. 2002, 2003; Xu et al. 2002; Haas et al. 2007).

Differences between strains in mechanical stimuli associated with blood flow and pressure would certainly influence gene expression in control arteries as well as flow-mediated collateral growth and gene expression. However, we have previously observed the increases in collateral flow and wall shear rate in this model to be similar between these two strains (Tuttle et al. 2002b). We have also demonstrated that the impairment of collateral growth in the SHR is independent of hypertension; the effect of agents on reversal of collateral growth impairment in SHR is more dependent upon antioxidant than antihypertensive properties (Miller et al. 2007a). Thus, we do not consider differences in pressure or collateral blood flow to be the primary cause of impaired collateral growth or abnormal gene expression in the SHR.

It is important to note that flow-mediated remodeling is mediated by multiple, complex mechanisms and the specific mechanisms responsible for impairment of flow-mediated remodeling may differ depending upon specific risk factors present (Kinnaird et al. 2008) and genetic background (Hochberg et al. 2002; Sheridan et al. 2007; Ceyhan et al. 2012). In this regard, while the WKY is typically used as the normotensive control for the SHR, genetic differences exist between these two inbred strains. Consequently, differences in collateral growth capacity and gene expression could result from the genetic differences between these strains. Thus, these results in SHR may not be representative for other conditions or rat strains including other models of hypertension. However, aging results in a similar impairment of flow-mediated remodeling (Miyashiro et al. 1997; Tuttle et al. 2002a) where a fundamentally different expression pattern in collaterals is observed at the protein level (Tuttle et al. 2002a). Thus, our current and previous (Tuttle et al. 2002a) studies suggest that abnormal transcriptional or translational mechanisms are involved in the impairment of collateral growth that may be associated with risk factors that include hypertension and aging.

Interpretation of microarray analysis of heterogeneous tissues can be difficult and has significant limitations. Expression changes which occur in one cell type may not be apparent if similar changes do not occur in other cell types or may be masked by changes in the opposite direction. Assessment of potential networks and pathways and interconnections can lead to incorrect conclusions because expression changes may be occurring in different cells. Also, differences in the baseline state can confound conclusions. In the current study, the samples were a heterogeneous mixture of the various cells normally present in the arterial wall. It is likely that differences in relative proportions existed between control arteries of WKY and SHR. However, our within-subject design comparing same animal collaterals to controls provided some control for such baseline genetic differences in the control arteries. Our network analyses are presented primarily to emphasize the overall difference in the gene expression patterns and indicate potential significance. The pathways are presented as areas where further investigation is warranted. Indeed, previous microarray studies in a similar tissue type (Dai and Faber 2010) and even more diverse tissues/organs (Lee et al. 2004; Packham et al. 2009) have demonstrated the ability of heterogeneous tissues to identify specific molecules, functional groups, and pathways which mediate collateral growth.

### Fundamental difference in gene expression patterns

#### Potential cause of impaired collateral growth

The enlargement of preexisting arteries which form alternative or collateral perfusion pathways and experience increased blood flow and shear stress after arterial occlusion is a complex process involving many coordinated cellular and molecular processes (Wahlberg 2003; Simons 2005). Changes in gene and protein expression of molecules regulating cell cycling, growth, development, and migration are associated with successful collateral growth (Lee et al. 2004; Dai and Faber 2010). Dai and Faber demonstrated that hindlimb collaterals from eNOS null mice exhibited reduced collateral growth and cellular proliferation and reduced expression of the majority of the upregulated cell cycle genes (Dai and Faber 2010). Our data in SHR with abnormal mesenteric NO regulation (Zhou et al. 2008) and impaired collateral growth (Tuttle et al. 2002b) are consistent with this observation, but indicate a potentially more global and fundamental difference in gene expression. While a similar number of genes had altered expression in collaterals of WKY and SHR, there was very little similarity or overlap in the specific genes altered (Fig. 2, Tables 4 and 5). This impacted not only groups of genes associated with cell cycling and proliferation but essentially every network, cellular, and molecular function, and canonical pathway examined (Fig. 2, Tables 1–3). Such global differences in gene expression are consistent with our previous proteomic study in aged rats (Tuttle et al. 2002a) and suggest that collateral growth impairment results from an abnormality in a fundamental process or regulatory mechanism that occurs at a level between signal transduction and gene transcription.

#### Potentially important molecules and canonical pathways

The comparison of global gene expression changes between WKY and SHR control and collateral arteries reveals many differences that may be of importance in advancing our understanding of the promotion and impairment of flow-mediated outward remodeling. It is not possible to discuss all of these within the scope of this paper. Altered molecules that may influence vascular growth, cell proliferation, and inflammation include CX3CL1 (fractalkine, neurotactin) (Green et al. 2006; Ryu et al. 2008; Borghese and Clanchy 2011), ESM1 (endocan) (Bechard et al. 2001; Béchard et al. 2001; Aitkenhead et al. 2002; Sarrazin et al. 2006; Rennel et al. 2007), CD74, HLA-DR (Schirmer et al. 2009; Borghese and Clanchy 2011; Fan et al. 2011), and GADD45A (Zhan et al. 1994; Bruemmer et al. 2005). Potentially relevant canonical pathways include mitochondrial dysfunction and signaling pathways involving nitric oxide (NO), TGFβ, and the renin–angiotensin system. Mitochondrial dysfunction may occur in the SHR (Graham et al. 2009; Piotrkowski et al. 2009) and has been linked to impaired vascular compensation for repetitive ischemia and reperfusion in the heart of obese Zucker rats (Pung et al. 2012). While the specific enzymatic and cellular sources of NO remain controversial (Dai and Faber 2010; Troidl et al. 2010), the majority of available evidence supports a substantial role for NO in flow-mediated remodeling and collateral growth. Abnormal NO signaling is observed (Maffei et al. 2002; Zhou et al. 2008), and flow-mediated, NO dilation is impaired (Qui et al. 1994; Matrougui et al. 1997) in SHR mesenteric arteries. Consistent with these observations, our work has shown that in vivo NO production is not increased with flow in SHR mesenteric arteries but that basal NO levels are greatly elevated by abnormally high concentrations of hydrogen peroxide (Zhou et al. 2008). The latter observation is consistent with reports of increased eNOS activity and/or elevated NO production in SHR (Kelm et al. 1995; Nava et al. 1995, 1998; Qiu et al. 1998; Briones et al. 2000; Maffei et al. 2002). While eNOS and iNOS expression and inhibition have been the primary end points evaluated in NO-dependent collateral growth, the abnormal regulation discussed above and the altered expression of multiple molecules within the cardiovascular NO signaling pathway of WKY (Fig. 3) suggest that a more comprehensive analysis is needed to understand the role of this important signaling pathway in collateral growth.

The renin–angiotensin system may be involved in both the promotion and impairment of vascular compensation to arterial occlusion (Silvestre et al. 2002; Tamarat et al. 2002) but its specific role in the outward remodeling of collateral arteries has not been examined. Our observations of AGT downregulation (Tables 4 and 8), differential regulation of the AGTR1 in WKY and SHR collaterals (Table 8, Fig. 5), and prevention of collateral growth by losartan in the WKY (Fig. 6) suggest an important but potentially complex role for this important signaling pathway. The AGTR1 may be activated by increased stretch or distension (Yasuda et al. 2008) such as would occur with the dilation of collateral arteries. AGTR1 expression can be downregulated by NO (Ichiki et al. 1998) which is elevated in the SHR mesenteric arteries (Maffei et al. 2002; Zhou et al. 2008). Interestingly, AGTR1 expression is also elevated selectively during pregnancy in the uterine arteries which experience elevated blood flow (Bird et al. 1997) and recent clinical studies support the potential significance of this important signaling system in collateral growth (Ahimastos et al. 2006; Ceyhan et al. 2012).

#### Potential abnormalities in transcriptional regulation

Among the molecules with the greatest fold changes in SHR control arteries were a number of downregulated molecules that are involved in transcriptional control (Table 6C). Identification of predicted transcription factor binding sites within the genes with altered collateral expression also demonstrated a fundamental difference between WKY and SHR (Table 7). Lists of both downregulated transcription regulators in SHR control arteries (Table 6C) and transcription factors with predicted binding sites in molecules altered in WKY but not SHR collaterals (Table 7) included transcription factors known to be influenced by both mechanical forces and redox status. Redox status can have a profound effect on transcription factor regulation and gene expression as reviewed by Allen and Tresini (Allen and Tresini 2000) and the SHR mesenteric arteries are characterized by an abnormal redox state with significant elevation in both NO and H_2_O_2_ (Maffei et al. 2002; Zhou et al. 2008). NFĸB is stimulated by shear stress in endothelial cells, may have a requisite role in flow-mediated outward remodeling, and is sensitive to oxidative and nitrosative stress (Lan et al. 1994; Khachigian et al. 1995; Flescher et al. 1998; Du et al. 1999; Grumbach et al. 2005; Castier et al. 2009; Chen et al. 2010). Our data demonstrated NFĸB nuclear translocation in WKY and apocynin-treated SHR collaterals which enlarge successfully, but not in collaterals of untreated SHR characterized by impaired collateral growth (Fig. 5). We also demonstrated a functional requirement for NFkB in successful collateral growth (Fig. 5C). Our studies have shown that apocynin restores a normal redox status (Zhou et al. 2008), NFĸB nuclear translocation (Fig. 5), and collateral growth capacity in the SHR (Miller et al. 2007a). The specific mechanisms responsible for the inhibition of NFĸB nuclear translocation in response to elevated shear stress in the SHR warrant more investigation and may involve NO-dependent mechanisms such as p50 nitrosylation (Matthews et al. 1996; Grumbach et al. 2005), downregulation of NFĸB1 and therefore p50, or inhibition by elevated H_2_O_2_ as observed in human T cells (Flescher et al. 1998) at concentrations similar to those we have measured in the SHR mesenteric arteries (Zhou et al. 2008). Such inhibition of activation and/or decreased expression of NFĸB components could mediate, at least in part, the abnormal expression pattern we observed in SHR collaterals in this study as well as the impairment of outward remodeling and cellular proliferation (Tuttle et al. 2002b). Regardless of the specific molecules involved, the present data support the hypothesis that chronic redox changes in the vasculature alter the transcriptional regulation that occurs in response to mechanical stimuli such as shear stress in collateral arteries subsequent to arterial occlusion.

### Conclusion and Clinical Significance

As reviewed by Ziegler et al. (2010), clinical observations indicate a remarkable capacity for vascular compensation to peripheral arterial occlusion which is profoundly compromised in the presence of vascular disease risk factors. The flow-mediated dilation and enlargement of preexisting small arteries provide the primary compensation to focal arterial occlusion in rodent and human limbs (reviewed by Ziegler et al. 2010). The enlargement of these vessels is impaired in the presence of vascular risk factors, but the specific mechanisms responsible are unknown and represent important targets for novel therapies. Such therapies to promote vascular growth and function are needed to prevent the progression of peripheral arterial disease (Gornik 2009). Significant evidence has accumulated that oxidative stress and a proinflammatory state are associated with impaired flow-mediated remodeling and collateral growth in animals and humans (Vita et al. 2008; Ziegler et al. 2010) and that antioxidant therapy reverses this impairment. Our results support the hypothesis that abnormal transcription factor activation and gene expression mediate, at least in part, the impairment of collateral growth in the SHR. Based upon our recent observations of abnormal H_2_O_2_ and NO in SHR and aged collaterals (Zhou et al. 2008, 2009), and the abnormal collateral gene (current study) and protein (Tuttle et al. 2002a) expression, we further hypothesize that redox status modulates shear-mediated gene expression in collateral arteries. Thus, therapies to correct an imbalance between reactive nitrogen and oxygen species in the presence of vascular disease risk factors could be important either as primary or adjuvant treatment to enhance collateral development.
